# Sexually Dimorphic Effects of CYP2B6 in the Development of Fasting-Mediated Steatosis in Mice: Role of the Oxylipin Products 9-HODE and 9-HOTrE

**DOI:** 10.3390/biomedicines13020295

**Published:** 2025-01-25

**Authors:** Jazmine A. Eccles-Miller, Tyler D. Johnson, William S. Baldwin

**Affiliations:** Biological Sciences, Clemson University, Clemson, SC 29634, USA; eccles@clemson.edu (J.A.E.-M.); tdj2@clemson.edu (T.D.J.)

**Keywords:** cytochrome P450, oxylipins, steatosis, fasting-induced steatosis, sexually dimorphic

## Abstract

**Background**: Cytochrome P450 2B6 (CYP2B6) is a sexually dimorphic, anti-obesity CYP enzyme responsible for the metabolism of xeno- and endobiotics, including the metabolism of polyunsaturated fatty acids (PUFAs) into 9-hydroxyoctadecadienoic acid (9-HODE) and 9-hydroxyoctadecatrienoic acid (9-HOTrE). However, humanized CYP2B6 transgenic (hCYP2B6-Tg) mice are sensitive to diet-induced hepatic steatosis despite their resistance to obesity. The purpose of this study was to determine if 9-HODE, 9-HOTrE, or other factors contribute to the sexually dimorphic steatosis observed in hCYP2B6-Tg mice. **Results:** Cyp2b9/10/13-null (Cyp2b-null) mice were injected with either 9-HODE or 9-HOTrE for 2 days and were then subjected to a fasting period of 20 h to induce steatosis. Serum lipids were moderately increased, especially in females, after 9-HODE (triglycerides (TGs), very low-density lipoproteins (VLDLs)) and 9-HOTrE (high-density lipoproteins (HDLs), low-density lipoproteins (LDLs), cholesterol) treatment. No change in hepatic lipids and few changes in hepatic gene expression were observed in mice treated with either oxylipin, suggesting that these oxylipins had minimal to moderate effects. Therefore, to further investigate CYP2B6’s role in steatosis, hCYP2B6-Tg and Cyp2b-null mice were subjected to a 20 h fast and compared. Both male and female hCYP2B6-Tg mice exhibited increased steatosis compared to Cyp2b-null mice. Serum cholesterol, triglycerides, HDLs, and VLDLs were increased in hCYP2B6-Tg males. Serum triglycerides and VLDLs were decreased in hCYP2B6-Tg females, suggesting the greater hepatic retention of lipids in females. Hepatic oxylipin profiles revealed eight perturbed oxylipins in female hCYP2B6-Tg mice and only one in males when compared to Cyp2b-null mice. RNA-seq also demonstrated greater effects in females in terms of the number of genes and gene ontology (GO) terms perturbed. There were only a few overlapping GO terms between sexes, and lipid metabolic processes were enriched in hCYP2B6-Tg male mice but were repressed in hCYP2B6-Tg females compared to Cyp2b-nulls. **Conclusions:** hCYP2B6-Tg mice are sensitive to fasting-mediated steatosis in males and females, although the responses are different. In addition, the oxylipins 9-HODE and 9-HOTrE are unlikely to be the primary cause of CYP2B6’s pro-steatotic effects.

## 1. Introduction

Obesity is a growing issue worldwide, and the World Health Organization has estimated that nearly 1 in 8 people are obese [1]. Hepatic steatosis is a common comorbidity with obesity and may lead to metabolic dysfunction, insulin resistance, and type 2 diabetes [2]. The combination of obesity, hepatic steatosis, and extrahepatic dysfunction is referred to as metabolic dysfunction-associated fatty liver disease (MAFLD) [3]. Liver diseases are particularly important to study and understand, given that the liver is responsible for the allocation of nutrients, the metabolism of xenobiotics, and regulating metabolism [4,5].

Recent research demonstrates that the cytochrome P450 2B (CYP2B) family is associated with obesity. Mice express five Cyp2b family members, three of which are highly expressed in the liver and potentially protective from obesity. Cyp2b9/10/13-null (Cyp2b-null) mice experience diet-induced obesity with significant steatosis, with males being much more susceptible than females [6]. Furthermore, CYP2B6 is protective against diet-induced obesity in female transgenic mice expressing the human CYP2B6 gene (hCYP2B6-Tg), with males showing greater steatosis surprisingly coupled with greater glucose resistance, as measured with a glucose tolerance test [7]. Similarly to mice, CYP2B6 is the only human CYP2B family member in which reduced expression is associated with increased body mass index [8].

There are several potential mechanisms by which CYP2B6 could be an anti-obesity enzyme that provides protection against insulin resistance while increasing steatosis [7]. CYP2B6 is a key detoxication enzyme that also metabolizes polyunsaturated fatty acids (PUFAs), steroids, and bile acids [9,10]. The disruption of any of these pathways could increase obesity as increased levels of PUFAs, steroid hormones, and bile acids are associated with obesity [11,12,13]. CYP2B6 metabolizes PUFAs to a variety of potential signaling molecules called oxylipins [14], most likely under high-fat diet conditions [7,15,16].

For example, CYP2B6 activity is inhibited by several PUFAs such as docosahexaenoic acid (DHA), arachidonic acid (AA), linoleic acid (LA), and α-linolenic acid (ALA) [10]. Each of these PUFAs are also substrates and produce multiple oxylipins with preferential metabolism in the 9- > 13-position. 9-hydroxyoctadecadienoic acid (9-HODE) from LA and 9-hydroxyoctadecatrienoic acid (9-HOTrE) from ALA are two of the most prominently produced oxylipins under high-fat diets in vivo and in vitro conditions, respectively [7]. 9-HODE liver concentrations ranged from approximately 8.3 μM to 6.4 μM in male and female mice, respectively [17]. 9-HOTrE liver concentrations are much lower (0.7–2 nM), presumably due to the difference in LA and ALA concentrations in the diet [7]. Other enzymes may also contribute to the production of these oxylipins. For example, 9-HODE is also produced by cyclooxygenase (COX) and lipoxygenase (LOX) enzymes [18]. Ultimately, oxylipins have a variety of roles, and some may serve as local or endocrine messengers that modulate inflammation, cell growth, pain, differentiation, and energy metabolism, which are therefore of interest in lipid use and obesity.

9-HODE is produced from LA-exposed CYP2B6 baculosomes in vitro, and high-fat diet-treated CYP2B6-Tg mice produced significantly more 9-HODE than high-fat diet-treated Cyp2b-null mice in vivo [7]. 9-HOTrE is preferentially produced more than any other oxylipin from ALA-exposed CYP2B6 baculosomes in vitro; however, 9-HOTrE was not measured in vivo [7]. This was most likely due to the high quantities of LA in the 60% high-fat diet and the relatively low amount of ALA present in the diet, causing LA to outcompete ALA as a substrate [7]. 9-HODE and 9-HOTrE are also of primary interest for CYP2B6-mediated steatotic activity because they increase hepatic lipid accumulation and the expression of genes associated with lipid uptake and synthesis in HepG2 hepatocarcinoma cells [19]. Therefore, we hypothesized that these oxylipins participate in the altered hepatic lipid accumulation observed in hCYP2B6-Tg mice.

The roles of 9-HODE and 9-HOTrE are not well studied. 9-HODE increases lipid accumulation through the activation of peroxisome proliferator-activated receptor (PPAR) γ2 in murine marrow-derived UAMS-33 cells [20] and also increases lipid uptake in monocytes [21]. 9-HODE also participates in inflammatory pain response signaling through the activation of transient receptor potential vanilloid type 1 (TRPV1) [18]. 9-HODE and 9-HOTrE activated murine and human PPARα; however, only murine PPARγ was activated [7]. There are very few studies on 9-HOTrE action. 9-HOTrE increased lipid accumulation and adipocyte differentiation in murine-derived 3T3-L1 fibroblasts [18], but no mechanism was proposed. Its activation of murine PPARγ may play a role.

Given that 9-HODE and 9-HOTrE are preferentially produced by CYP2B6 and alter hepatic lipid accumulation and gene expression in vitro, we hypothesized that these oxylipins are contributing to the increased steatosis seen in hCYP2B6-Tg mice. Traditional high-fat diet studies are time-consuming and costly, with high-fat diet treatment lasting approximately 10–25 weeks and costing thousands in animal care alone. To avoid long-term treatment with oxylipins, exposure to oxylipins was performed over the course of 2 days, and on the second day of treatment, mice were fasted for 20 h to induce steatosis. Fasting-mediated steatosis has been used successfully in mice to study a variety of pathways related to steatosis, including the involvement of the bile acid receptor (24 h fast) and the role of increased fatty acid oxidation in skeletal muscle (24 h fast) [22,23]. Fasting protocols ranging from 4 to 72 h have been investigated in mice, and hepatic triglyceride accumulation is significantly evident at 16 h [24]; therefore, the 20 h fasting time was chosen based on this 16–24 h range. This presents a unique opportunity to complete short-term exposure to 9-HODE or 9-HOTrE and evaluate their effects on the development of steatosis and its subsequent effects on serum lipids while avoiding expensive long-term treatment. Therefore, to investigate the effects of 9-HODE or 9-HOTrE on the development of steatosis, we performed intraperitoneal injections of either 9-HODE, 9-HOTrE, or a vehicle control in Cyp2b-null mice. To further investigate the role of CYP2B6 in the development of steatosis in vivo and compare the effects of individual oxylipins with CYP2B6 activity in mice, hCYP2B6-Tg mice were also subjected to a 20 h fasting period to investigate the development of steatosis compared to Cyp2b-null mice.

We used a fasting protocol to study the role of CYP2B6, 9-HODE, and 9-HOTrE in steatosis. Cyp2b-null mice were treated with 9-HODE and 9-HOTrE because this model produces lower levels of these oxylipins [7]. We hypothesized that 9-HODE and 9-HOTrE would contribute to fasting-mediated hepatic steatosis and alter the expression of genes involved in lipid metabolism, uptake, and synthesis in a manner similar to hCYP2B6-Tg mice in comparison to Cyp2b-null mice using RNAseq. Similarities observed between 9-HODE/9-HOTrE-treated Cyp2b-null mice compared to hCYP2B6-Tg and Cyp2b-null fasted mice would be interpreted as indicators that 9-HODE/9-HOTrE are a significant reason for the increased steatosis in hCYP2B6-Tg mice. Differences observed between the oxylipin-treated and hCYP2B6-Tg mice would be interpreted as evidence that other factors besides these oxylipins are driving CYP2B6-mediated steatosis.

## 2. Materials and Methods

### 2.1. Care and Genotyping of Cyp2b-Null and hCYP2B6-Tg Mice

Animal care and use protocols were approved by Clemson University’s Institutional Animal Care and Use Committee (AUP 2019-061). Cyp2b9/10/13-null (Cyp2b-null; C57BL/6J-Del(7Cyp2b10-Cyp2b9)^Fatso/mmnc^, https://www.mmrrc.org/catalog/sds.php?mmrrc_id=50703 (accessed on 17 January 2025)) [25] and humanized CYP2B6-transgenic (hCYP2B6-Tg; 71652; https://www.mmrrc.org/catalog/sds_temp_new.php?mmrrc_id=71652 (accessed on 17 January 2025)) mice on a Cyp2b-null background [26,27], both generated by our lab, are available from the NIH-sponsored Mouse Mutant Resource and Research Centers. DNA was isolated from ear punches to perform genotyping using the QuantaBio (Beverly, MA, USA) AccuStart II Genotyping kit according to the manufacturer’s instructions, as described previously [26].

### 2.2. Treatment of Cyp2b-Null Mice with 9-HODE or 9-HOTrE

For 9-HODE and 9-HOTrE treatments, both male and female Cyp2b-null 10–12-week-old mice (*n* = 4–5) were injected with either 9-HODE or 9-HOTrE at 1 mg/kg in saline or with saline alone as a vehicle control once per day for 2 days. Following the day 2 injection, mice were fasted for 20 h and necropsies were performed. The 9-HODE treatment dosage was chosen based on physiologically relevant concentrations of 9-HODE seen in the liver [7], and the 9-HOTrE treatment dosage was chosen to mirror the concentrations of 9-HODE given. Treatment with oxylipins for 2 days was chosen to encompass the 20 h period of fasting, as well as a day’s worth of meals to show the potential repression of fasting-mediated steatosis.

### 2.3. Fasting of Cyp2b-Null and hCYP2B6-Tg Mice

To compare fasting-mediated steatosis between Cyp2b-null and hCYP2B6-Tg mice, 10–12-week-old male and female Cyp2b-null and hCYP2B6-Tg mice (*n* = 4–5 per sex and genotype) were fasted for 20 h prior to euthanasia and necropsy.

### 2.4. Necropsy

Mice were weighed prior to euthanasia. Mice were then anesthetized under 3% isoflurane; blood was collected by heart puncture and then they were euthanized by CO_2_ asphyxiation, as confirmed by bilateral pneumothorax. Whole blood was collected via heart puncture. Liver was excised, weighed, and cut into several sections for Oil-Red O staining, triglyceride extraction, lipid extraction and analysis, and RNA extractions. Most tissues were snap-frozen in liquid nitrogen for storage at −80 °C.

### 2.5. Serum Lipid Markers

Blood was incubated at room temperature for 30 min and centrifuged at 6000 rpm for 10 min to isolate serum. Serum was transferred into a new microcentrifuge tube and snap-frozen in liquid nitrogen prior to storage at −80 °C. Aliquots were sent to Baylor College of Medicine’s Comparative Pathology lab (Houston, TX, USA) to be evaluated for serum lipids including cholesterol, triglycerides (TGs), high-density lipoproteins (HDLs), low-density lipoproteins (LDLs), and very low-density lipoproteins (VLDLs) with a Beckman–Coulter AU480 analyzer (Beckman Coulter, Brea, CA, USA), according to the manufacturer’s instructions (*n* = 4–5).

### 2.6. Liver Lipids and Lipidomics

Liver sections from the mice were sent to Baylor College of Medicine’s Comparative Pathology laboratory for Oil Red O staining of frozen sections, according to standard procedure [28], to visualize lipids. Several images of Oil Red O-stained sections were taken using Leica Acquire software Version 3.4.7 Build 9121 (Wetzlar, Germany) and evaluated with ImageJ 1.53i (Laboratory for Optical and Computational Instrumentation, Madison, WI, USA) using a previously tested method to quantify the lipid droplet area [26]. A separate section of liver was extracted, and triglycerides were quantified colorimetrically according to the manufacturer’s instructions (Cayman Chemical Co., Ann Arbor, MI, USA) at an absorbance rate of 530 nm on a BioTek Synergy H1 hybrid spectrophotometer (US BiotTek Laboratories, Shoreline, WA, USA).

Lipidomic analysis for oxylipins from liver samples was performed by the Emory Integrated Metabolomic and Lipidomic Core (EIMLC) for the analysis of metabolites from AA, LA, ALA, and DHA. Oxidized lipids were selectively extracted from samples by solid-phase extraction following EIMLC protocols [29]. Oxylipins were extracted and enriched from liver homogenates by solid-phase extraction because of their low abundance. Approximately 50 mg of the liver sample was homogenized, suspended in methanol acidified to a pH of 3.0, placed onto the C18 solid-phase extraction cassette, rinsed with hexane, and eluted with methyl formate using an automated robot (Biotage, Uppsala, Sweden). The enriched oxylipin sample was then analyzed by liquid chromatography–tandem mass spectrometry on a Sciex QTrap5500 (Framingham, MA, USA) using a quantitative multiple reaction monitoring-based method that is highly selective. A Thermo Accucore C18 column (4.6 × 100 mm, 2.6 μm) (Thermo Fisher Scientific, Waltham, MA, USA) provided the chromatographic resolution of the oxylipins using an 18 min stepwise gradient using solvent A as water (with 1 mM ammonium formate) and solvent B as acetonitrile (with 1 mM ammonium formate). The flow rate was 0.5 mL/min at a column temperature of 50 °C, and the gradient worked as follows: 0–0.5 min, 10% A; 0.5–1.0 min, 10% to 50% A; 1.0–2.0 min, 50% A; 2.0–2.1 min, 50% to 75% A; 2.1–5.0 min, 75% A; 5.0–7.0 min, 75% to 85% A; 7.0–13.0 min, 75% A; 13.0–14.0 min, 85% to 10% A; 14.0–18.0 min, 10% A. Ten μL of the extracted sample was injected. Instrumental parameters—collision energy (−70 arbitrary units), declustering potentials (−50 V), electrospray voltage (−4500 V), and temperature (65 °C)—were optimized with synthetic standards and held consistent over the course of analysis. Samples were analyzed in the negative ion mode by using unit mass resolution. Data processing for oxylipins was conducted using Sciex proprietary software Multiquant 1.7.3 (Sciex, Framingham, MA, USA), whereby the area under the curve for each sample was calibrated against external standards for the quantification of analytes in the liver.

### 2.7. RNA Sequencing (RNAseq)

Total RNA was extracted with Trizol reagent and quantified using a Nanodrop (Thermo Fisher Scientific). Aliquots were sent to Novogene (Sacramento, CA, USA) for library preparation, and mRNA was purified from total RNA using poly-T oligo-attached magnetic beads fragmented to synthesize cDNA from the poly-A tail enrichment. The samples were sequenced with a paired-end 150 NovaSeq 6000 system (Novogene, Sacramento, CA, USA) to an average of 47,782,986 raw reads per sample. Raw reads were processed by Novogene using their in-house perl scripts. Clean reads were then aligned to the murine reference genome (GCF_000001635.25_GRCm38.p6) using Hisat2 v2.0.5, and featureCounts v1.5.0-p3 was used to count the mapped reads. Series GSE282292, containing the RNAseq data, has been uploaded to the gene expression omnibus (GEO).

Differential gene expression analysis was performed by Novogene Inc. with the DESeq2 R package (1.20.0). The *p*-values from DESeq2 were then adjusted using Benjamini and Hochberg’s approach to control for false discovery rates. Genes with an adjusted *p*-value < 0.05 were considered differentially expressed genes (DEG) and were used for further analysis. Changes in pathways were determined using the Kyoto Encyclopedia of Genes and Genomes (KEGG) pathway analysis, and differences in gene ontology (GO) were determined with the Database for Annotation, Visualization, and Integrated Discovery (DAVID) [30,31]. Reduce and Visualize Gene Ontology (Revigo) [32] was used to visualize differences in GO terms between the models or after oxylipin treatment.

### 2.8. Quantitative PCR

qPCR was performed using primers specific for *Gapdh*, *18S*, *Ppp1r3c*, *Lipc*, *Lpin1*, *Igfbp2*, *Plscr4*, *Scd1*, and *Plin4*. Primer sequences (idtDNA, Coralville, IA, USA) and annealing temperatures can be found in Table 1. For qPCR, 1 μL cDNA was mixed with 12.5 μL RT^2^SYBR Green ROX qPCR Mastermix (Qiagen, Frederick, MD, USA), 9.5 μL molecular biology grade water, 1 μL forward primer, and 1 μL reverse primer, resulting in a final volume of 25 μL per well. All plates were heated to 95 °C for 1 min, followed by 50 cycles of denaturation at 95 °C for 30 s, annealing for 30 s, and elongation for 45 s at 72 °C, then followed by a melt curve starting at 55 °C for 5 s up to 95 °C for 5 min.

Efficiency was determined using a standard curve consisting of a mix of samples diluted from 1:1 to 1:1024. Protocols were carried out, and fluorescence was measured using a Bio-Rad CFX96 Real-Time System (Bio-Rad, Hercules, CA, USA). Gene expression was normalized to the geometric mean of *Gapdh* and *18S* as housekeeper genes, and changes in gene expression were quantified using the inverted Muller’s equation [33,34].

### 2.9. Hierarchical Clustering

Hierarchical clustering was performed using Morpheus software, available at https://software.broadinstitute.org/morpheus/ (accessed on 1 October 2024), courtesy of the Broad Institute of MIT and Harvard (Cambridge, MA, USA). Data such as RNAseq, oxylipins, serum, and liver lipids for each individual mouse were compiled and inserted into Morpheus to generate a heat map for each genotype or oxylipin treatment group.

### 2.10. Statistical Analysis and Graph Preparation

Statistical analyses were carried out using GraphPad Prism software 7.0 (San Diego, CA, USA). Significance was determined by unpaired *t*-tests to determine differences between groups. Graphing was performed on GraphPad Prism. Heat maps and hierarchical clustering were performed using Morpheus software (Cambridge, MA, USA). Data used in this manuscript are available in Supplementary Files and GEO—GSE282292.

## 3. Results and Discussion

### 3.1. 9-HODE and 9-HOTrE Increase Serum Lipids with Greater Effects in Females than Males

Male and female Cyp2b-null mice were treated with the mouse/human PPARα and mouse PPARγ activators [7], 9-HODE or 9-HOTrE, or a vehicle control at 1 mg/kg/day for 2 days and then fasted for 20 h. No differences in organ weight or gross anatomical differences were found between the control and treated mice (Supplementary Files S1 and S2). However, serum lipid levels increased following oxylipin treatment, with greater sensitivity in female mice than male mice. Female mice treated with 9-HODE displayed increased serum TGs and VLDLs by nearly 40% (Table 2). Females treated with 9-HOTrE displayed increased serum cholesterol, HDLs, and LDLs by 25–36% (Table 3); the three parameters not effected by 9-HODE. In contrast, males treated with 9-HODE displayed only a 26% increase in LDLs, and 9-HOTrE caused no significant changes in serum lipids (Table 3).

### 3.2. Oxylipins Did Not Perturb Liver TGs

**Figure 1:**
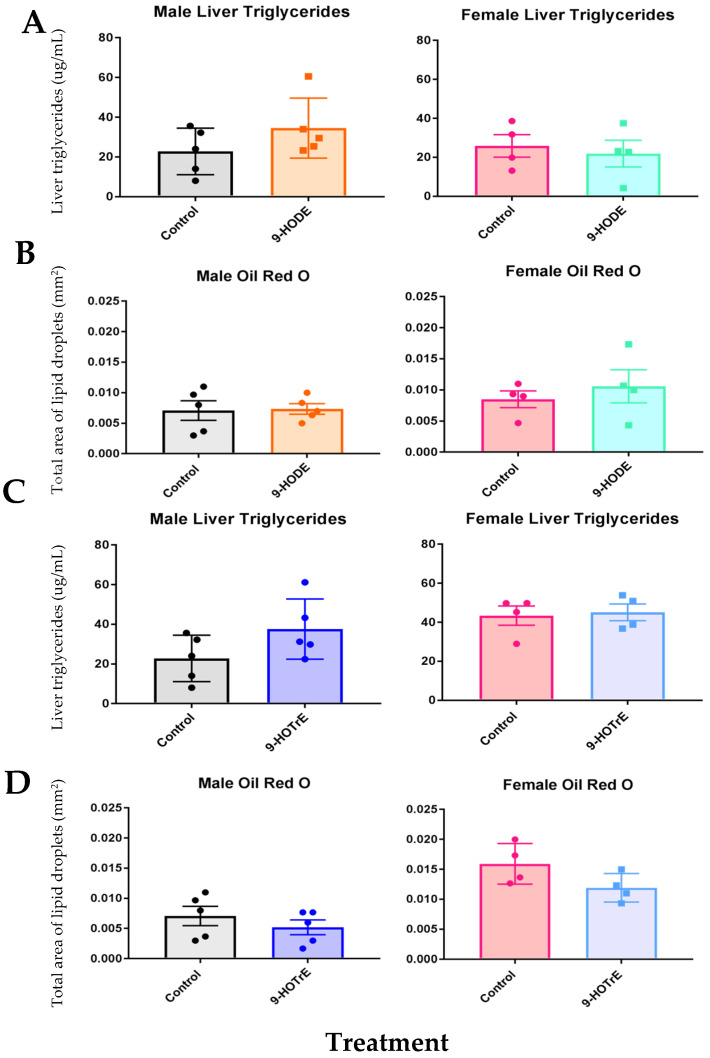
Quantification of liver triglycerides in 9-HODE- and 9-HOTrE-treated mice. (**A**) Quantification of male and female liver triglycerides colorimetrically after 9-HODE treatment. (**B**) Quantification of male and female liver triglycerides via Oil Red O staining of liver sections after 9-HODE treatment. (**C**) Quantification of male and female liver triglycerides colorimetrically after 9-HOTrE treatment. (**D**) Quantification of male and female liver triglycerides via Oil Red O staining of liver sections after 9-HOTrE treatment. Data are presented at mean ± SEM. Statistical significance was determined by unpaired *t*-tests using GraphPad Prism 7.0. No differences were observed.

Neutral lipids from treated mice were measured using a colorimetric assay and confirmed by Oil Red O staining (measures neutral lipids such as cholesterol and TGs), as described in the Materials and Methods (Figure 1). Neither sex showed differences in liver lipid content after 9-HODE or 9-HOTrE treatment in comparison to vehicle-treated controls, indicating that neither 9-HODE nor 9-HOTrE had significant effects on liver steatosis in Cyp2b-null mice. This is surprising given that 9-HODE and 9-HOTrE increased TGs in HepG2 hepatocarcinoma cells in the presence of the monounsaturated fat, oleic acid. However, under normal cell culture conditions, 9-HODE and 9-HOTrE did not increase steatosis [19]. Therefore, it is possible instead that steatosis 9-HODE and 9-HOTrE are inducing the clearance and transport of lipids based on the increase in serum lipids, especially in females (Table 2 and Table 3). This suggests the possibility that these oxylipins are increasing lipid production but not steatosis in mice [7,19].

### 3.3. Gene Expression Differences with Oxylipin Treatment

**Figure 2:**
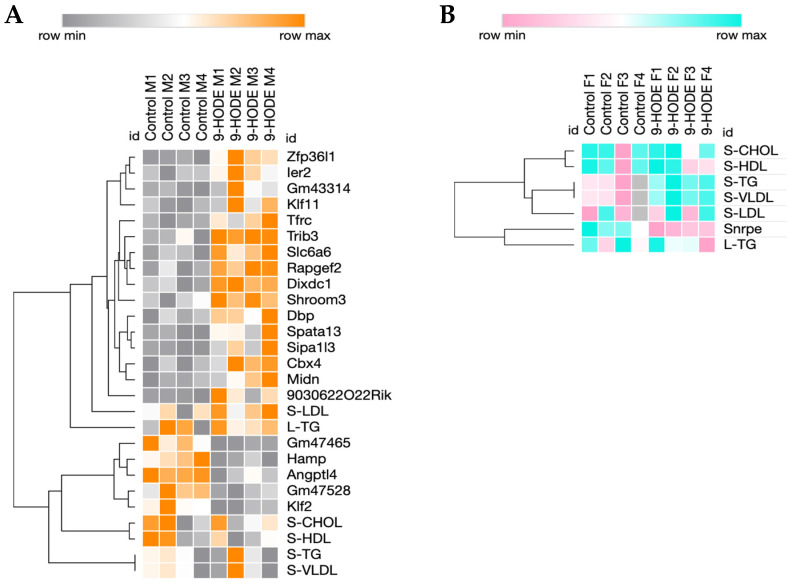
Hierarchical clustering of DEG and serum and liver lipids from male or female mice treated with 9-HODE. (**A**) Clustering of male data showed few associations of DEG with serum or liver lipid content. (**B**) Clustering of female data showed no associations between DEG and lipid content.

RNAseq was performed on liver tissue to estimate the ontologies and pathways altered by 9-HODE (Supplementary File S3) or 9-HOTrE (Supplementary File S4). DEG analysis showed few gene expression changes after the oxylipin treatments. 9-HODE treatment resulted in only twenty-one DEG in males and one DEG in females (Figure 2). Due to the low number of DEG in both sexes, GO term and KEGG pathway analysis showed no significance.

Hierarchical clustering showed few but weak associations between DEG and serum/liver lipids in males only (Figure 2). This was expected, with only one serum lipid being significantly altered in the males (LDL) and only one differentially expressed gene in the females following 9-HODE treatment. Interestingly, the genes weakly associated with serum lipids in males, *Angptl4*, *Hamp*, *Klf2*, *Gm47528*, and *Gm47465*, are all involved in lipid processing. For example, Angiopoietin-like 4 (*Angptl4*) inhibits lipoprotein lipase and therefore increases serum TGs [35]. Hepcidin (*Hamp*) is involved in iron regulation and is positively associated with non-alcoholic fatty liver disease (NAFLD) [36]. Kruppel-like factor 2 (*Klf2*) is a transcription factor that represses PPARγ and inhibits adipogenesis [37]. Predicted gene 47528 (*Gm47528*) is increased in hepatic Cpt1a-null male mice after fasting [38], and quantitative trait loci mapping for predicted gene 47465 (*Gm47465*) associates it with blood glucose and obesity [39]. Most of these genes are involved in or are regulated by PPARs and are positively associated with obesity or serum lipids; instead, these genes are repressed by 9-HODE in males as serum TGs and VLDLs go up (Figure 2).

Due to the low number of DEG observed in the liver but the increases observed in serum lipid levels, particularly in females, 9-HODE likely does not lead to any significant liver changes. 9-HODE is produced at high amounts by several enzymes, including the lipoxygenase (LOX) and cyclooxygenase (COX) enzymes, and the additional treatment of 9-HODE to Cyp2b-null mice may have made little difference to the overall 9-HODE concentrations.

**Figure 3:**
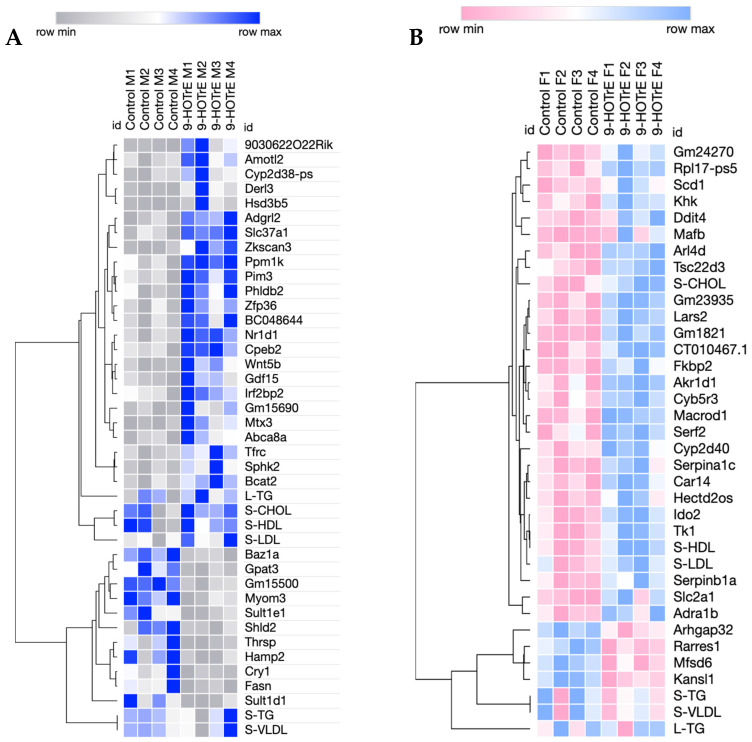
Hierarchical clustering of DEG and serum and liver lipids in male or female mice treated with 9-HOTrE. (**A**) Clustering of male data showed no close associations of gene expression changes and alterations in serum or liver lipids. (**B**) Clustering of female data showed associations between serum HDLs (S-HDLs) and LDLs (S-LDLs) with several genes.

9-HOTrE had a greater effect on hepatic gene expression than 9-HODE; however, the DEG analysis still showed few perturbed genes. Male mice treated with 9-HOTrE had 35 DEG and female mice treated with 9-HOTrE had 30 DEG (Figure 3). Due to the low number of DEG, there were no significant KEGG pathways identified. The only significant GO term identified was the “lipid metabolic process”, which was down-regulated in the 9-HOTrE-treated males. Genes of interest within the lipid metabolic process GO term category include fatty acid synthase (*Fasn*), branch chain aminotransferase 2 mitochondrial (*Bcat2*), glycerol-3-phosphate acyltransferase 3 (*Gpat3*), hydroxy-delta-5-steroid dehydrogenase, 3 beta- and steroid delta-isomerase 5 (*Hsd3b5*), sphingosine kinase 2 (*Sphk2*), sulfotransferase family 1D, member 1 (*Sult1d1*), sulfotransferase family 1E, member 1 (*Sult1e1*), and thyroid hormone responsive (*Thrsp*). The hierarchical clustering of male DEG with serum and liver lipids parameters showed no associations between the two sets of data (Figure 3A). However, none of the serum nor hepatic lipids were significantly perturbed (Table 3), making associations with DEG unlikely.

However, the hierarchical clustering of female DEG with serum and liver lipids showed an association between serum HDLs and LDLs with several genes. HDLs, LDLs, and cholesterol were increased by 9-HOTrE in females (Table 3). Genes that cluster with HDLs and LDLs include thymidine kinase 1 (*Tk1*), indoleamine 2,3-dioxygenase 2 (*Ido2*), carbonic anhydrase 14 (*Car14*), and serine (or cysteine) peptidase inhibitor clade a member 1c and clade b member 1a (*Serpina1c* and *Serpinb1a*). *Tk1* is an enzyme involved in the biosynthesis of dTTP for DNA replication and has been implicated in the progression of hepatocellular carcinoma [40]. *Ido2* is involved in the metabolism of tryptophan and is associated with inflammation in the liver [41]. *Car14* is predicted to be involved in one-carbon metabolism and has recently been shown to participate in the biliary bicarbonate buffering system, and Car14-knockout mice show greater liver damage compared to their wildtype counterparts following the ligation of the common bile duct [42]. *Serpina1c* and *Serpinb1a* are both implicated in inflammatory pathways within the liver [43,44]. Cholesterol directly clustered with the Glucocorticoid-Induced Leucine Zipper Protein (*Tsc22d3*), which is stimulated by glucocorticoids and acts as an anti-inflammatory signal [45], and ADP-ribosylation factor 4D (*Arl4d*), which buffers HDL cholesterol levels [46]. Gene expression changes coupled with increases in serum cholesterol, HDLs, and LDLs suggest that 9-HOTrE could increase hepatic inflammation in female mice. Overall, 9-HOTrE had a greater effect than 9-HODE, and this suggests that the lipid type we ingest (n-6 vs. n-3) is crucial in CYP2B6’s physiological actions.

Other genes such as *Cyb5r3*, *Macrod1*, *Serf2*, *Akr1d1*, *Fkbp2*, *Lars2*, and *Cyp2d40* also clustered with the greater clade associated with LDLs and HDLs. Several of these genes are associated with mitochondrial function or lipid metabolism (fatty acid, bile acid, and steroids) as well as NAFLD progression. These genes include leucyl-tRNA synthetase 2 (*Lars2*, mitochondrial protein production) [47], aldo-keto reductase family 1, member D1 (*Akr1d1*, bile acid and steroid metabolism) [48], cytochrome P450 family 2, subfamily d, polypeptide 40 (*Cyp2d40*, steroid and cholesterol metabolism) [49], mono-ADP Ribosylhydrolase 1 (*Macrod1*, enhances steroid function at receptor) [50], and cytochrome.

B5 Reductase 3 (*Cyb5r3*, fatty acid and cholesterol desaturation and elongation) [51]. *Cyb5r3* represses the benefits of caloric restriction [52]. Other genes perturbed by 9-HOTrE in female mice that are related to energy metabolism include stearoyl-CoA desaturase 1 (*Scd1*, fatty and cholesterol metabolism) [53] and ketohexokinase (*Khk*, metabolizes fructose) [54].

Overall, 9-HOTrE treatment in females led to the increased expression of genes involved in energy metabolism and inflammation, and some of the inflammatory genes clustered with HDLs and LDLs (Figure 3 and Table 3). In addition, serum cholesterol clustered with an increase in *Tsc22d3* (Table 3 and Figure 3). In summary, alterations in serum lipids levels with 9-HODE or 9-HOTrE treatment coupled with few gene expression changes in the liver may not be the only organ or primary organ affected by 9-HODE and 9-HOTrE.

### 3.4. Changes in Gross Anatomy and Serum Lipids Between hCYP2B6-Tg and Cyp2b-Null Mice Are Sexually Dimorphic

9-HODE and 9-HOTrE are some of the most highly perturbed oxylipins by CYP2B6 metabolism [7]. However, they had moderate to minimal effects on hepatic steatosis and gene expression. Therefore, we went back to our Cyp2b-null and hCYP2B6-Tg mice to confirm that hCYP2B6-Tg mice are sensitive to fasting-mediated hepatic steatosis (not just diet-induced), determine if oxylipins are associated with fasting-mediated steatosis, and compare the gene expression effects of 9-HODE and 9-HOTrE to the effects of adding CYP2B6 to the liver.

Male and female Cyp2b-null and hCYP2B6-Tg mice were fasted for 20 h, and body weight, organ weights, and serum and liver lipids measured. Female hCYP2B6-Tg mice weighed significantly less at the outset of the study compared to their Cyp2b-null counterparts, but saw no difference in organ weight between groups, leading to an increase in the liver-to-body weight ratio by 8.84% (Supplementary File S1). Male hCYP2B6-Tg mice had a significant increase in liver weight by 9.29%, but no other significant changes in gross anatomy were observed (Supplementary File S2).

Serum lipid analysis revealed strong sexually dimorphic differences between male and female hCYP2B6-Tg mice. Male hCYP2B6-Tg mice were highly sensitive to a 20 h fasting period, where they exhibited an increase in serum cholesterol, triglycerides (TGs), HDLs, and VLDLs compared to Cyp2b-null mice (Table 4). In contrast, female hCYP2B6-Tg mice exhibited a decrease in serum triglycerides and VLDLs compared to Cyp2b-null mice (Table 4). In summary, the presence of human CYP2B6 altered serum lipids with a strong sexually dimorphic response, where males show greater serum lipids and females lower serum TGs and VLDLs.

### 3.5. Liver TG Difference Between hCYP2B6-Tg and Cyp2b-Null Mice

**Figure 4:**
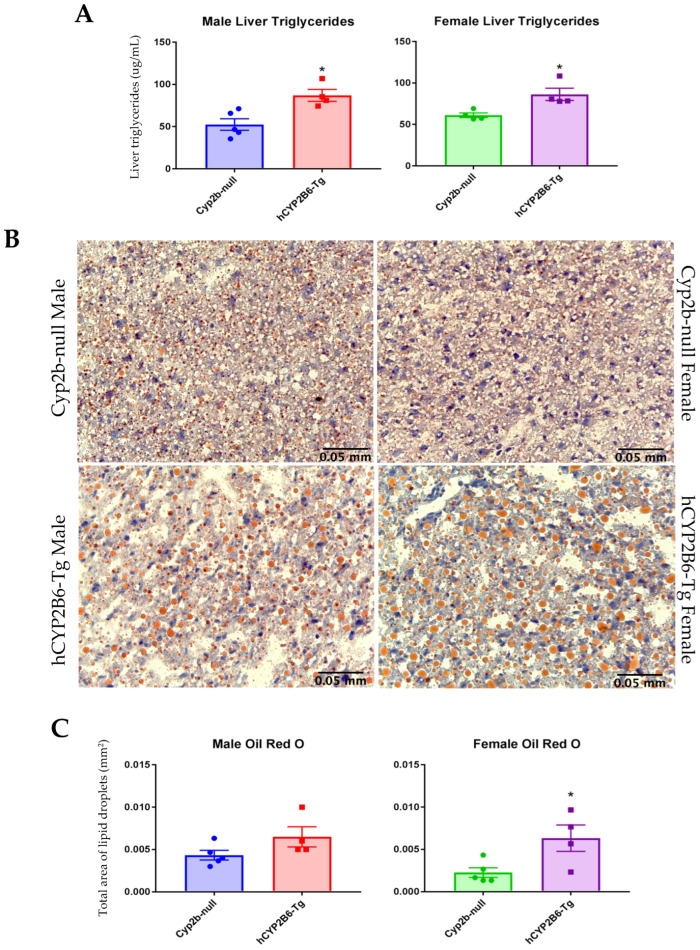
Increased hepatic triglyceride content in hCYP2B6-Tg mice. (**A**) Both male and female hCYP2B6-Tg mice show a significant increase in liver triglycerides measured colorimetrically. Quantification (**C**) of Oil Red-O-stained histological sections of liver (**B**) showed significance only in female hCYP2B6-Tg mice. Data are represented as mean ± SEM. Statistical significance determined by unpaired *t*-tests on GraphPad Prism 7.0. * *p*-value < 0.05.

Liver lipids were analyzed using a colorimetric TG assay and verified with Oil Red O staining and quantification. hCYP2B6-Tg mice exhibited increased liver TG content compared to Cyp2b-null mice in both sexes (Figure 4A). Oil Red O staining and quantification confirmed a significant increase in TGs in females (Figure 4B,C). Males exhibited a similar trend with both the colorimetric assay and Oil Red O staining; however, only the colorimetric assay was significant (Figure 4). The increased release of lipids from the liver into the serum in males may explain the lack of statistical significance in Oil Red O measurements coupled with increased serum lipids.

Females showed a significant increase in lipid colorimetric and Oil Red O staining coupled with reduced serum lipids, indicating the preferential retention of lipids in the liver. Males have greater serum and liver lipid levels although liver TG levels may be somewhat muted. Overall, these data suggest a sexually dimorphic response in which they have lower serum lipids because of liver lipid retention. RNAseq was performed in part to determine the potential mechanisms leading to increased retention.

### 3.6. Gene Expression Differences Between hCYP2B6-Tg and Cyp2b-Null Mice

RNAseq was performed on liver tissues to evaluate differences in gene expression between Cyp2b-null and hCYP2B6-Tg mice and provide insight on CYP2B6-mediated hepatic steatosis. Analysis of differentially expressed genes (DEG) showed a total of 807 DEG between Cyp2b-null and hCYP2B6-Tg males and 1224 DEG between Cyp2b-null and hCYP2B6-Tg females (Table 5). Earlier studies demonstrated that these mouse models have similar liver gene expression profiles under normal diet/control conditions [27], indicating that they respond differently under fasting or dietary stress conditions. In addition, 152 DEG were shared between sexes, 96 of these genes were dysregulated similarly by each sex, and 56 genes were dysregulated but in an opposing manner (Table 5). Both the Cyp2b-null and hCYP2B6-Tg mice responded vigorously to fasting based on hepatic steatosis and gene expression changes. Furthermore, a clear sexually dimorphic response occurred under fasting conditions in hCYP2B6-Tg mice, highlighting the importance of CYP2B6 expression differences in males and females and how it may influence lipid homeostasis. A full list of DEG and GO/KEGG analysis can be found (Supplementary Files S5–S9).

### 3.7. Gene Expression Changes Evaluated via qPCR

To confirm the data previously elucidated by the RNA sequencing data above, qPCR was performed to confirm specific DEG involved in lipid utilization by RNAseq. Protein phosphatase 1 regulatory subunit 3C (*Ppp1r3c*), insulin-like growth factor binding protein 2 (*Igfbp2*), and phospholipid scramblase 4 (*Plscr4*) were not differentially expressed in female mice by RNAseq, only in males. qPCR confirmed significant expression changes in five of the seven genes evaluated in male mice and three of the four genes differentially expressed by RNAseq in female mice (Table 6). All of the DEG by RNAseq trended in the same direction as the qPCR data, except Perilipin 4 (*Plin4*) in females. Overall, RNAseq and qPCR showed very similar results.

The genes analyzed by qPCR are associated with processes involving lipid metabolism, homeostasis, and transport, except for *Igfbp2*, which mediates insulin-like growth factor (IGF) activity and half-life [55]. *Ppp1r3c* strongly up-regulates glycogen synthesis in hepatocytes [56]. Lipin-1 (*Lpin1*) and *Plscr4* are involved with lipid transport and mobilization [57], and hepatic lipase (*Lipc*) catalyzes the formation of the serum carrier molecule and LDL cholesterol via the production of hepatic lipase [58]. *Scd1* codes for the stearoyl-CoA desaturase enzyme that produces monounsaturated fatty acids from saturated fats [59], and *Plin4* encodes for a lipid-coating protein that protects lipid droplets from endogenous lipases [60]. These data verify the RNAseq data and confirm sexually dimorphic changes in gene expression.

### 3.8. GO Term and KEGG Pathway Analysis of Cyp2b-Null vs. Hcyp2b6-Tg DEG

**Figure 5:**
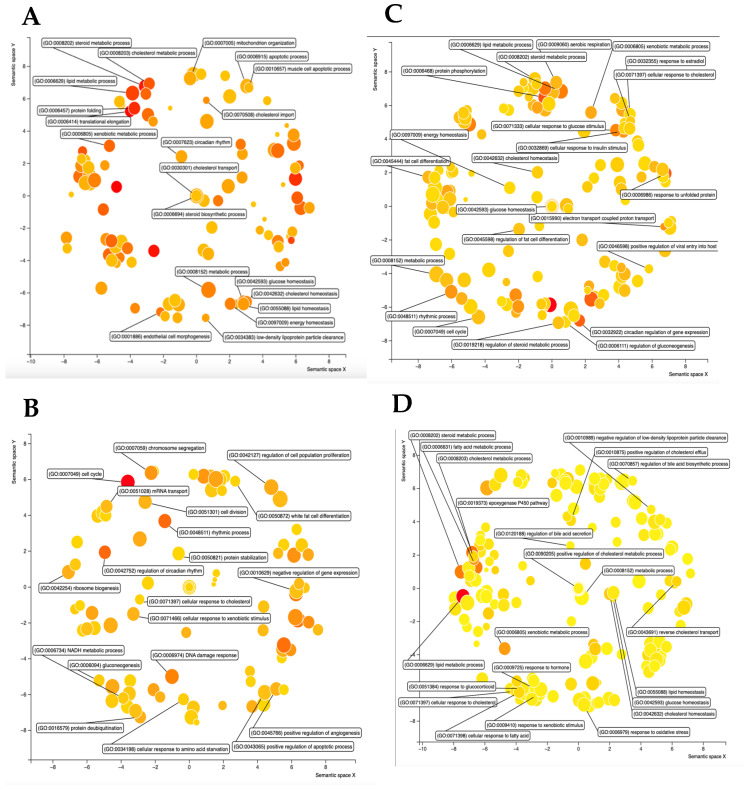
Gene ontology (GO) terms in the Biological Processes category enriched in either hCYP2B6-Tg mice or Cyp2b-null mice. (**A**) GO terms enriched in hCYP2B6-Tg male mice. (**B**) GO terms enriched in Cyp2b-null male mice. (**C**) GO terms enriched in hCYP2B6-Tg female mice. (**D**) GO terms enriched in Cyp2b-null female mice. GO terms were included if they had an FDR (adjusted *p*-value) of <0.05. Larger dots indicating greater number of genes and darker colors (reds) indicates greater statistical significance.

Gene ontology (GO) was performed and Revigo was used to visualize changes in GO terms in the “biological processes” category. Male hCYP2B6-Tg mice displayed a variety of enriched GO terms related to lipid and steroid metabolism (Figure 5A); terms enriched in male Cyp2b-null mice included those related to cell cycle and circadian rhythm (Figure 5B). GO terms enriched in female hCYP2B6-Tg mice included the circadian regulation of gene expression as well as several terms related to proteins, such as response to unfolded protein, protein catabolic process, and protein phosphorylation (Figure 5C). Terms enriched in female Cyp2b-null mice included lipid metabolic process, cholesterol metabolic process, and steroid metabolic process (Figure 5D). Genes involved in lipid and steroid metabolic processes responded in opposing directions in the male and female hCYP2B6-Tg mice: enriched in male hCYP2B6-Tg mice and conversely enriched in female Cyp2b-null mice (Figure 5). Given the increase in hepatic steatosis in the female hCYP2B6-Tg mice, the enrichment of lipid, cholesterol, steroid, and fatty acid metabolic processes as the top four enriched terms in Cyp2b-null mice may help explain the reduced steatosis as an adaptive response.

**Figure 6:**
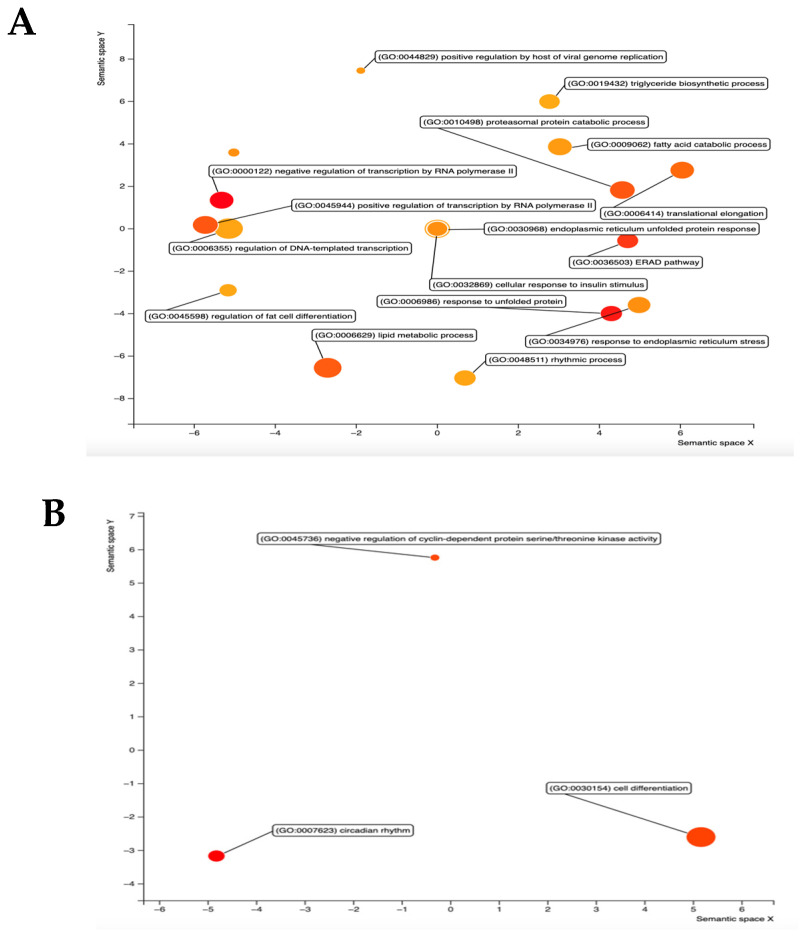
Visualization of GO terms shared between male and female DEG analysis. Significant DEG were used to generate GO term lists across sexes that were not dysregulated in an opposing manner. (**A**) GO terms enriched in hCYP2B6-Tg mice. (**B**) GO terms enriched in Cyp2b-null mice. Shared GO terms were identified within these lists then visualized using Revigo. GO terms included have a *p*-value of <0.05. Larger dots indicating greater number of genes and darker colors (reds) indicates greater statistical significance.

Interestingly, GO term analysis of shared up- and down-regulated genes by sex, excluding genes dysregulated in a contradicting manner, showed the lipid metabolic process as an enriched term in hCYP2B6-Tg mice. Other terms enriched in hCYP2B6-Tg mice include the regulation of transcription from RNA pol II promoter, response to unfolded protein, and response to ER stress (Figure 6). Sex-shared GO terms enriched in Cyp2b-null mice include circadian rhythm and the negative regulation of cyclin-dependent protein serine/threonine kinase activity (Figure 6). Supplementary Files S5–S9 found in Mendeley Data (https://data.mendeley.com/datasets/wnvkz8f7ft/1 (accessed on 31 December 2024)), which provides a full list of GO terms with corresponding Revigo figures.

DEG analysis was also used to perform KEGG pathway analysis to determine the pathways affected by dysregulated genes. KEGG pathways enriched in male hCYP2B6-Tg mice include protein processing in endoplasmic reticulum, steroid hormone biosynthesis, complement and coagulation cascades, and the metabolic pathway (Supplementary File S5). Pathways enriched in male Cyp2b-null mice included Hippo signaling pathways, hepatocellular carcinoma, breast cancer, and AGE-RAGE signaling pathway diabetic complications (Supplementary File S6).

Pathways enriched in female hCYP2B6-Tg mice included phosphatidylinositol signaling pathway, prostate cancer, inositol phosphate metabolism, and oxidative phosphorylation (Supplementary File S7). Pathways enriched in female Cyp2b-null mice included chemical carcinogenesis–DNA adducts, drug metabolism, other enzymes, steroid hormone biosynthesis, the biosynthesis of cofactors, and peroxisome (Supplementary File S8).

In summary, hCYP2B6-Tg mice showed large changes in gene expression compared to Cyp2b-null mice, and many of these changes were sexually dimorphic. Female mice were more sensitive to fasting, as they showed a greater number of DEG (Table 5). The enrichment of GO terms associated with lipid metabolism was sexually dimorphic, as male hCYP2B6-Tg mice showed the enrichment of terms related to lipid metabolism and female Cyp2b-null mice often showed the enrichment of similar terms (Figure 5). When evaluating shared DEG between sexes, the GO term “lipid metabolic process” was enriched in hCYP2B6-Tg mice (Figure 6), showing that both sexes did experience alterations in gene expression related to lipid metabolism, but male hCYP2B6-Tg mice had a greater number of genes associated with this term. Female hCYP2B6-Tg mice showed a greater number of terms related to proteins, suggesting that males experience more changes in lipid metabolism in the liver under fasting conditions and that females experience greater changes in protein function and metabolism under fasting conditions. A ranked list of GO and KEGG terms enriched in either hCYP2B6-Tg or Cyp2b-null male and female mice is provided in Supplementary Files S5–S9.

### 3.9. Oxylipin Profile Changes Between hCYP2B6-Tg and Cyp2b-Null Mice

**Figure 7:**
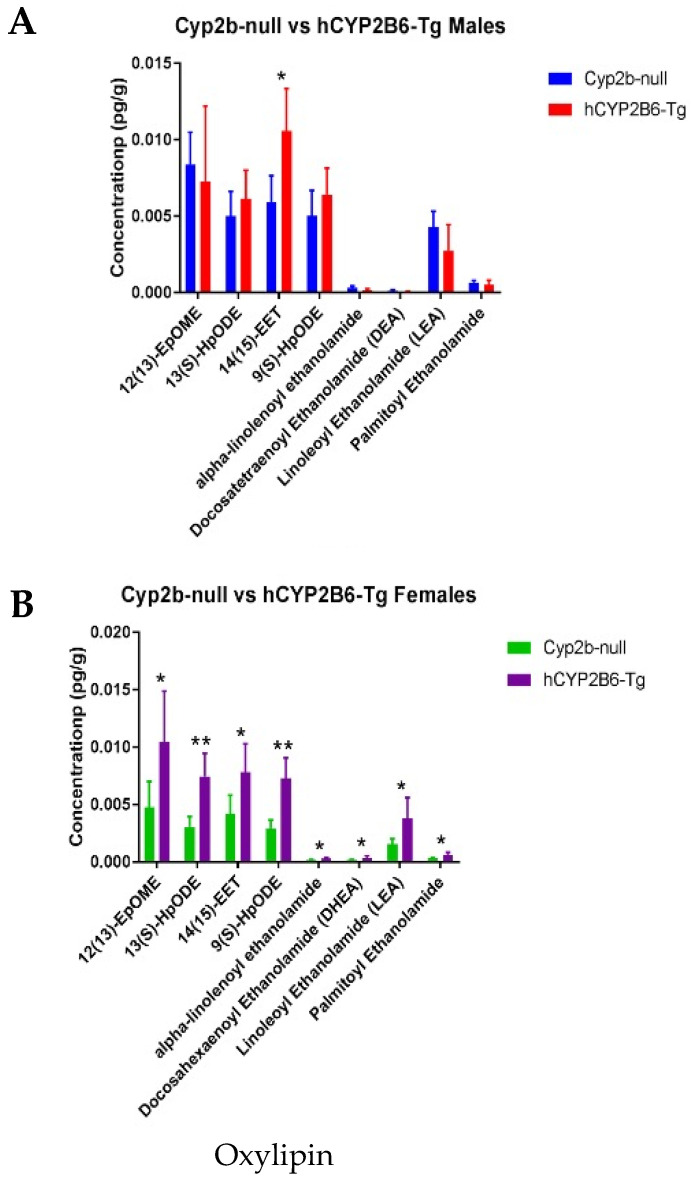
Oxylipin profiles in Cyp2b-null vs. gCYP2B6-Tg mice. (**A**) Profiles showed increased concentrations of only one oxylipin in livers of hCYP2B6-Tg male mice. (**B**) Profiles showed increased concentrations of eight oxylipins in livers of in hCYP2B6-Tg female mice. Multiple *t*-tests. Mean ± SEM. * *p* value < 0.05, ** *p* value < 0.01.

Oxylipins were quantified using selective solid-phase extraction and analyzed by liquid chromatography–mass spectrometry to determine differences between genotypes. 14,15-epoxyeicosa-5,8,11-trienoic acid (14(15)-EET) was the only oxylipin increased in male hCYP2B6-Tg mice (Figure 7A), and this AA-derived oxylipin was also increased in females (Figure 7B). Female hCYP2B6-Tg also had several other oxylipins with increased liver concentrations, including 9-hydroperoxyoctadecadienoic acid (9(S)-HpODE), 13-hydroperoxyoctadecadienoic acid (13(S)-HpODE), docosahexaenoyl ethanolamide (DHEA), linoleoyl ethanolamide (LEA), 12(13)epoxy-9Z-octadecenoic acid (12(13)-EpOME), palmitoyl ethanolamide, and alpha-linolenoyl ethanolamide (Figure 7B). 9-HpODE and 13-HpODE are precursors to 9-HODE and 13-HODE [14]. 12(13)-EpOME and 14(15)-EET are also produced by CYP2B6 in vitro and in vivo [7], and both oxylipins were increased in female hCYP2B6-Tg mice (Figure 7B). 14,15-EET has several established functions, including suppressing mitochondrial apoptosis during ischemic injury and several established functions within pulmonary endothelial cells [14]. Some of these oxylipins, such as 9-HpODE, 13-HpODE, and 12(13)-EpOME, are associated with oxidative stress [14].

14(15)-EET (AA metabolite) and 12(13)-EpOME (LA metabolite) are produced by CYP enzymes [14], and both are produced by CYP2B6 [7]. 9(S)-HpODE and 13(S)-HpODE (LA metabolites) can be produced by both the LOX and CYP enzymes [14,61], and they are also produced by CYP2B6 [7]. DHEA (DHA metabolite), LEA (LA metabolite), palmitoyl ethanolamide (palmitic acid metabolite), and alpha-linolenoyl ethanolamide (ALA metabolite) are members of a group of compounds referred to as N-acylethanolamines (NAEs) that are produced via the N-acylphosphatidylethanolamine (NAPE) pathway [62]. NAEs are lipid derivatives involved in signaling for satiety, mitochondrial energetics, stress, and a variety of other pathways [63,64]. The association of NAEs with metabolic diseases such as obesity and steatosis has drawn greater attention in recent years, with many of these metabolites showing anti-obesity effects [65,66,67]. Recent research has shown that fasting for periods between 3 and 24 h results in an increased level of NAEs in the liver and may be a response to oxidative stress and cross-talk between the brain and liver during periods of glucose deprivation [68], but the exact mechanism of this response is still under investigation.

### 3.10. Hierarchical Clustering of DEG with Oxylipins

**Figure 8:**
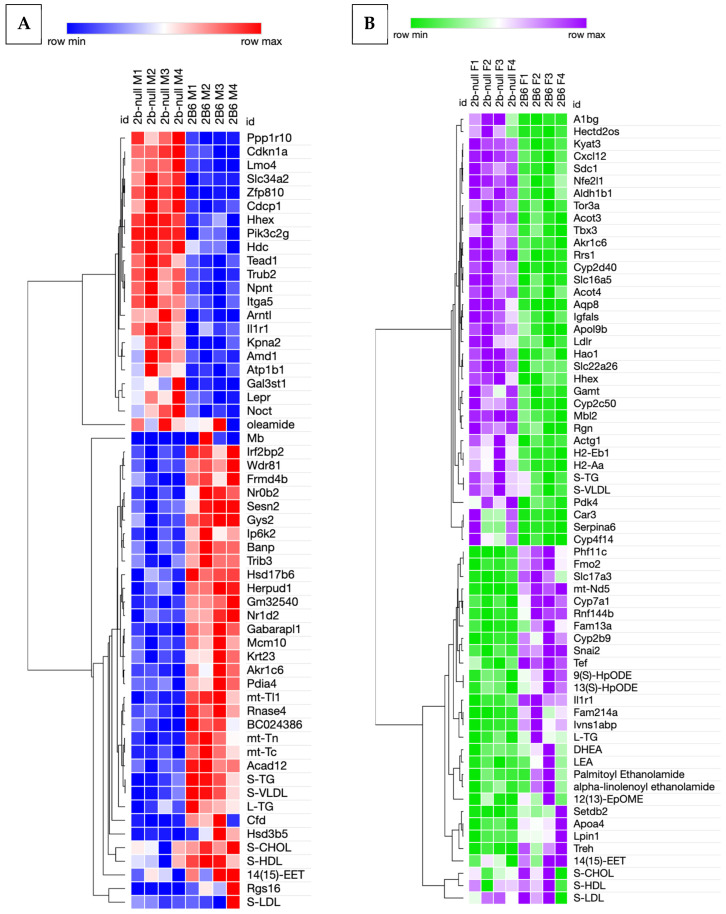
Hierarchical clustering of DEG, oxylipins, serum, and liver lipids. (**A**) Hierarchical clustering of differential gene expression data and oxylipins/lipids in male Cyp2b-null and hCYP2B6-Tg mice showed minimal co-clustering. (**B**) Hierarchical clustering of differential gene expression data and oxylipins/lipids comparing female Cyp2b-null and hCYP2B6-Tg mice showed that some oxylipins and serum lipids clustered with DEG.

Hierarchical clustering was performed to compare DEG to oxylipins and serum lipids. In males, few DEG and lipids co-clustered; however, DEG that did cluster with lipids are crucial in fatty acid oxidation. For example, serum LDLs clustered with Regulator of G protein Signaling 16 (*Rgs16*), which inhibits fatty acid oxidation in hepatocytes [69] by enhancing GTPase activity and repressing signal transduction (Figure 8). Serum TGs and VLDLs clustered with acyl-CoA dehydrogenase member 12 (*Acad12*), mitochondrially encoded tRNA asparagine (*Mt-Tn*), and mitochondrially encoded tRNA cysteine (*Mt-Tc*), and also, to a lesser extent, with mitochondrially encoded tRNA leucine (*Mt-Tl1*), ribonuclease 4 (*Rnase4*), and *BC024386*. *Acad12* is homologous to ACAD10 in humans, an enzyme that assists with fatty acid b-oxidation in the mitochondria [70]. *Mt-Tn*, *Mt-Tc*, and *Mt-Tl1* function as tRNAs [71]. *BC024386* translationally represses *Nlrp6* [72], an inflammasome gene expressed in the intestine and liver, which is important in repressing metabolic disease progression [73]. These data suggest that β-oxidation is disrupted along with increased TGs and VLDLs.

In females, the drop serum TGs and VLDLs clustered with reduced actin gamma 1 (*Actg1*), histone compatibility 2, class II antigen E beta (*H2-Eb1*), and histone compatibility 2, class II antigen A alpha (*H2-Aa*) (Figure 8). *Actg1* is involved in the synthesis of actin for the cytoskeleton, and increased expression has been associated with increased fibrosis [74,75]. Both *H2-Eb1* and *H2-Aa* are involved in antigen sensing [76,77]. Greater liver triglycerides in females were associated with a gene of unknown function, *Fam214a*, interleukin-1 receptor type 1 (*IL1r1*), and influenza virus NS1A binding protein (*Ivns1abp*) which encodes the Nd1 actin binding protein. All three of these genes have previously been associated with NAFLD [78,79,80,81], and two (*Il1r1*, *Fam214a*) are associated with inflammation. Thus, increased liver triglycerides were associated with NAFLD, increased inflammation, and reduced serum triglycerides with reduced antigen sensing genes.

In females, there are 17 genes in the larger clade associated with the oxylipins 9(S)-HpODE, 13(S)-HpODE, and 14(15)-EET (Figure 8); other differentially produced oxylipins were not closely associated with DEG. Of these seventeen genes, five are associated with fatty acid metabolic processes via GO term analysis. These include *Fmo2*, *Cyp7a1*, *Apoa4*, *Treh*, and *Lpin1* (Figure 8). 9(S)-HpODE and 13(S)-HpODE clustered with several genes, including *Cyp7a1*, involved in cholesterol and bile acid synthesis. *Cyp7a1* is protective against NAFLD and inflammation through the increased activation of FXR [82], and this gene was enriched in hCYP2B6-Tg female mice despite these mice experiencing increased steatosis (Supplementary File S6). Flavin containing dimethylaniline monoxygenase 2 (*Fmo2*) is a xenobiotic metabolizing enzyme inversely associated with NAFLD. The increased expression of *Fmo2* is associated with repressed Srebf1 activation and protection against steatosis [83]. This gene was enriched in hCYP2B6-Tg female mice despite increased steatosis (Supplementary File S6). *Apoa4* is involved in lipid transport and LDL oxidation, and increased expression is associated with reduced gluconeogenesis and protection against liver steatosis [84,85]. Trehalose (*Treh*) is an enzyme associated with cortisol or high-sugar diet-induced NAFLD [86]. *Lpin1* is involved in the synthesis of triglycerides and can act as a co-activator of both PPARα and γ, further influencing lipid metabolic pathways [87]. These associations indicate that CYP2B6, through the production of oxylipins, alters fatty acid homeostasis in the liver, although both genes associated and inversely associated with steatosis were increased in hCYP2B6-Tg mice.

Other genes that clustered closely with these oxylipins in females include a transcription factor involved in epithelial–mesenchymal transition (snail family transcriptional repressor 2 (*Snai2*)—protects from liver fibrosis) [88], a factor involved in thyroid-stimulating hormone (Thyrotroph embryonic factor (*Tef*)), a ubiquitin-protein transferase (Ring Finger Protein 144B (*Rnf144b*)), a mitochondrial NADH dehydrogenase (mitochondrially encoded NADH dehydrogenase 5 (*Mt-Nd5*); part of complex 1), a histone methyltransferase (SET Domain Bifurcated Histone Lysine Methyltransferase 2 (*Setdb2*)), and a GTPase protein (Family With Sequence Similarity 13 Member A (*Fam13a*)), which represses AMPK activity and is associated with fatty liver disease. Fam13a-null mice are resistant to high-fat diet induced obesity and NAFLD [89]. *Setdb2* has been purposely down-regulated (it is increased in hCYP2B6-Tg mice) to reduce NAFLD [90]. Interestingly, of the eight genes associated with NAFLD, four are closely associated with 14(15)-EET, and all four are associated with NAFLD.

The only oxylipin that was differentially produced in male hCYP2B6-Tg mice was 14(15)-EET. It clustered somewhat similarly to increased serum HDLs and cholesterol; however, there were no genes closely clustered to 14(15)-EET. Previous research showed 14(15)-EET is associated with HFD-induced steatosis in hCYP2B6-Tg mice [7]. However, other research indicates 14(15)-EET is inversely associated with NAFLD and non-alcoholic steatohepatitis (NASH) [91]. Further investigation of 14(15)-EET is needed to determine the importance of this metabolite.

Male and female hCYP2B6-Tg mice had different responses to fasting. Male hCYP2B6-Tg mice showed a significant increase in serum cholesterol, triglycerides, HDLs, and VLDLs compared to Cyp2b-null mice, and female hCYP2B6-Tg mice showed a decrease in serum triglycerides and VLDLs compared to Cyp2b-null mice (Table 3). Both male and female hCYP2B6-Tg mice showed increased steatosis compared to Cyp2b-null mice, and female mice showed a stronger steatotic response and more DEG than male mice (Figure 4), possibly due to the decreased serum lipid load. DEG varied significantly between sexes, with only 18.8% of total DEG between genotypes in males and 12.4% of DEG between genotypes in female mice being shared between sexes. Upon further evaluation of the shared genes, these 152 DEG were often dysregulated in a different direction between sexes. Additionally, 36.8% of these 152 genes showed dysregulation in different directions when comparing sexes, further emphasizing the sexual dimorphism of the liver, especially in hCYP2B6-Tg mice, a gene that is female-predominant [92].

These very different and sometimes contradictory transcriptomic responses between male and female mice emphasize the importance of evaluating steatotic responses in both sexes. CYP2B6 is sexually dimorphic [92], and the receptors primarily responsible for the regulation of CYP2B6 are also sexually dimorphic. The constitutive androstane receptor (CAR) is the strongest regulator of CYP2B6, and CAR regulation is part of a complex network that is sexually dimorphic. CAR is regulated by the hepatocyte nuclear factor 4 alpha (HNF4α), which in turn is regulated by the signal transducer and activator of transcription 5 a and b (STAT5A and STAT5B) via temporal patterns of growth hormone release from the pituitary, which differ in males and females [93]. CAR activity is also repressed by androgens [94,95], leading to the greater expression of genes regulated by CAR in females than males. Forkhead box protein A2 (FOXA2) also participates in the regulation of CYP2B6, and FOXA2 and STAT5B regulate a number of energy-related genes in male mice [96], further driving sexual dimorphism. Interestingly, CAR’s expression is circadian and many of the circadian rhythm genes, including GH-regulated igf-1, are sexually dimorphic [97]. We have previously demonstrated that hCYP2B6-Tg mice show sexually dimorphic responses to a high-fat diet as related to steatosis and obesity [7]; therefore, it was expected that some degree of sexual dimorphism would be present in the results of this study investigating fasting-mediated steatosis due to the upstream regulation of CYP2B6 and previously observed sex-influenced differences.

Transcriptomic analysis revealed that male Cyp2b-null mice were more sensitive to changes in circadian rhythm and cell cycle than male hCYP2B6-Tg mice. Female Cyp2b-null mice did not experience the same disruption of these processes. Instead, female hCYP2B6-Tg mice were more sensitive to changes in circadian rhythm. Female Cyp2b-null showed the enrichment of genes and processes involved in cholesterol, steroid, and fatty acid metabolism. Opposingly, hCYP2B6-Tg male mice showed the enrichment of genes and processes involved in cholesterol, steroid, and fatty acid metabolism. hCYP2B-Tg female mice showed the enrichment of processes related to protein phosphorylation and folding, xenobiotic response, and glucose and insulin responses, while several of these processes were enriched in male Cyp2b-null mice. These examples demonstrate the sex differences between the male and female mice when fasting and may indicate differences in the transport, synthesis, and metabolism of fatty acids that explain differences in serum lipids (Table 3).

For example, genes involved in lipid synthesis that are enriched in hCYP2B6-Tg males include *Lpin1* [98] and *Lpin2* [99]. Genes involved in lipid synthesis that are enriched in female hCYP2B6-Tg mice, including all three members of the lipin family (*Lpin1*, *Lpin2*, and *Lpin3*) [98,99,100], include diacylglycerol kinase Eta (*Dgkh*) [101], acetoacetyl-CoA synthetase (*Aacs*) [102], and acetyl-Coenzyme A carboxylase alpha (*Acaca*) [103]. Genes involved in lipid transport showed sexually dimorphic changes. Male hCYP2B6-Tg mice showed the enrichment of genes related to lipid transport, including kallikrein B1 (*Klkb1*) involved in HDL-mediated cholesterol efflux [104] and the D site of the albumin promoter binding protein (*Dbp*), which is crucial for fatty acid transport in the blood via its function in the transport protein albumin [105]. Female Cyp2b-null mice showed the enrichment of genes related to lipid transport, including several apolipoproteins associated with or predicted to be associated with HDLs and VLDLs (*Apom*, *Apol9a*, *Apol9b*) [106]. This provides a possible explanation for the increase in serum lipids seen in male hCYP2B6-Tg mice and the decrease in serum lipids seen in female hCYP2B6-Tg mice (Table 4). Genes involved in lipid metabolism enriched in male Cyp2b-null mice include carboxylesterase 1D (*Ces1d*) [107], glycerophosphodiester phosphodiesterase 1 (*Gde1*) [108], and *Scd1* [109]. Genes involved in lipid and fatty acid metabolism enriched in Cyp2b-null female mice include three of the five members of the acyl-CoA synthetase long-chain family member (*Acsl1*, *Acsl3*, and *Acsl5*) [110] and *Scd1* [109].

While this study identifies transcriptomic changes related to steatosis and CYP2B6 in vivo using transgenic mouse models, as well as physiological differences seen between Cyp2b-null and hCYP2B6-Tg mice by evaluations of lipidomics and serum and liver lipids, there are limitations. RNA expression does not always directly equate to protein expression or activity. However, this paper provides physiological outcomes, comparisons between the models and the two oxylipins produced by CYP2B6, and possible mechanisms to investigate more thoroughly for future studies on the interplay between toxicology, diet, and metabolic disease. Furthermore, other tissues may contribute to hepatic steatosis due to their misuse of fats. This study focused on the liver and serum, as that is where CYP2B6 is primarily expressed. In future studies, investigation of other tissues such as adipose and skeletal muscle may be warranted to further understand the whole-body response.

Fasting-induced steatosis has been used for a variety of applications [23,24,111] and proved useful in this study evaluating genotype and sex differences between Cyp2b-null and hCYP2B6Tg mice. Fasting-induced steatosis is also a much faster and cheaper option for studying fatty liver disease than diet-induced obese models. In this study, hCYP2B6-Tg mice experienced increased steatosis compared to Cyp2b-null mice, indicating that a 20 h fasting period is sufficient to observe substantial differences in steatosis. Substantial sexual dimorphism was also observed, and therefore, fasting-induced steatosis could be useful for understanding the differences in the metabolic response of the liver between males and females in genetic, diet-induced, and toxicology models.

## 4. Conclusions

**Figure 9:**
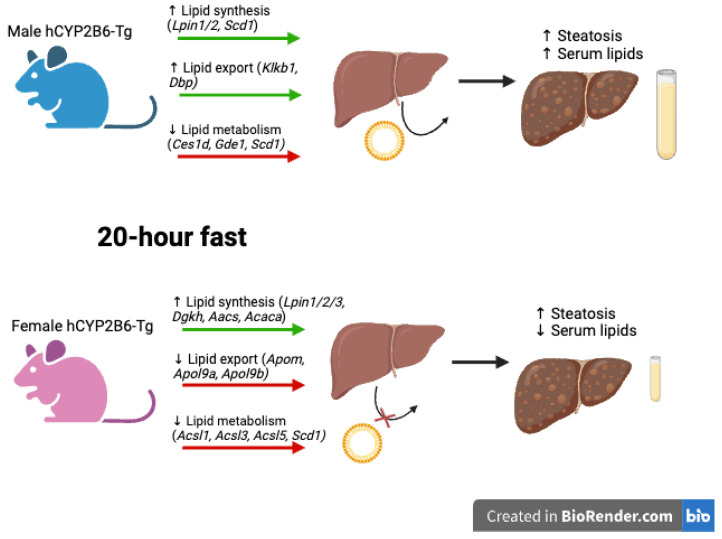
Summary of proposed sexually dimorphic lipid metabolic alterations caused by CYP2B6 in fasting-mediated steatosis. Comparisons of Cyp2b-null to hCYP2B6-Tg mice after a 20 h period of fasting indicated that male hCYP2B6-Tg mice appear to increase lipid synthesis in the liver and transport of lipids out of the liver while decreasing lipid metabolism, leading to increased steatosis and increased serum lipids. Female hCYP2B6-Tg mice in comparison to Cyp2b-null mice appear to increase lipid synthesis in the liver but decrease lipid metabolism and transport of lipids out of the liver, leading to increased steatosis but decreased serum lipids. Example genes are provided over the green (up-regulated pathways) and red arrows (down-regulated pathways). Figure created using BioRender.com.

CYP2B6 is a sexually dimorphic gene with greater expression in females [83]. Two of the metabolites synthesized by CYP2B6, 9-HODE from LA and 9-HOTrE from ALA, did not show significant effects on the livers of treated mice. Serum lipids were typically only affected in males. In general, 9-HOTrE, 9-HODE, and Cyp2b-null vs. hCYP2B6-Tg mice all showed sexually dimorphic effects (Figure 9). Female mice experienced a stronger steatotic response and a greater number of differentially expressed genes; male mice experienced greater changes in serum lipids coupled with less severe steatosis. Male hCYP2B6-Tg mice appear to show increased lipid metabolism and export compared to females, which were more likely to retain fats within the liver, leading to decreased serum lipids and greater steatosis. Female mice also showed more changes in their oxylipin profile than male mice, as female mice had increased concentrations of eight oxylipins, whereas male mice had only one. Overall, this study demonstrated that a 20 h fast can induce significant steatosis in hCYP2B6-Tg mice, CYP2B6 in vivo elicits a strong sexually dimorphic response in lipid metabolism and liver steatosis which affects females more than males, and the oxylipins 9-HODE and 9-HOTrE are only a minor part of CYP2B6’s effects, leaving other oxylipins or lipid metabolites that most likely mediate its anti-obesity and steatotic effects.

## Figures and Tables

**Table 1:** Primer sequences and annealing temperatures for qPCR.

Gene	Forward Sequence	Reverse Sequence	Annealing Temp (°C)
*Gapdh*	CATCACTGCCACCCAGAAGACTG	ATGCCAGTGAGCTTCCCGTTCAG	50
*18S*	AGTCCCTGCCCTTTGTACACA	CGATCCGAGGGCCTCACTA	56
*Ppp1r3c*	GCGTTGTGTTTGCTGACTCC	CGGTTGAAGGCTGAGGGAAAT	62.4
*Lipc*	CTTCCAGCCTGGCTGCCACTT	GCAAGGAGTCAATGAAGAGGTGC	60
*Lpin1*	TAAACGGAGCCGACACCTTGGA	CCGTTGTCACTGGCTTGTTTGG	60
*Igfbp2*	CCTCAAGTCAGGCATGAAGGAG	TGGTCCAACTCCTGCTGGCAAG	60
*Plscr4*	ATCCTGTGACGAATCAGCCTGC	GAGGCTCAACATGCTGAAGAACG	60
*Scd1*	GCAAGCTCTACACCTGCCTCTT	CGTGCCTTGTAAGTTCTGTGGC	60
*Plin4*	GCACTAAGGACACGGTGACCAC	GACCACAGACTTGGTAGTGTCC	60

**Table 2:** Serum lipid analysis in Cyp2b-null 9-HODE-treated mice.

Serum Parameter	Control Male	9-HODE Male
Cholesterol (mg/dL)	95.68 ± 4.733	97.23 ± 3.236
Triglycerides (mg/dL)	67.28 ± 2.832	64.75 ± 2.843
HDL (mg/dL)	59.44 ± 2.536	57.79 ± 1.597
LDL (mg/dL)	2.924 ± 0.2501	3.672 ± 0.2044 *
VLDL (mg/dL)	13.36 ± 0.5657	12.95 ± 0.5682
**Serum Parameter**	**Control Female**	**9-HODE Female**
Cholesterol (mg/dL)	73.08 ± 3.458	75.11 ± 1.789
Triglycerides (mg/dL)	47.5 ± 3.711	66.3 ± 2.012 **
HDL (mg/dL)	43.33 ± 2.105	43.11 ± 1.859
LDL (mg/dL)	3.763 ± 0.4153	4.128 ± 0.3377
VLDL (mg/dL)	9.503 ± 0.7429	13.26 ± 0.4023 **

Data are presented as mean ± SEM. Statistical differences determined by unpaired Student’s *t*-tests with GraphPad Prism 7.0. * *p*-value < 0.05, ** *p*-value < 0.01.

**Table 3:** Serum lipid analysis in Cyp2b-null 9-HOTrE-treated mice.

Serum Parameter	Control Male	9-HOT9-HOTrE Male
Cholesterol (mg/dL)	95.68 ± 4.733	102.4 ± 3.529
Triglycerides (mg/dL)	67.28 ± 2.832	63.23 ± 5.781
HDL (mg/dL)	59.44 ± 2.536	61.2 ± 1.995
LDL (mg/dL)	2.924 ± 0.2501	3.916 ± 0.3611
VLDL (mg/dL)	13.36 ± 0.5657	12.64 ± 1.156
**Serum Parameter**	**Control Female**	**9-HOTrE Female**
Cholesterol (mg/dL)	61.27 ± 3.395	83.57 ± 3.203 **
Triglycerides (mg/dL)	68.45 ± 5.27	62.1 ± 2.438
HDL (mg/dL)	29.92 ± 0.9884	37.61 ± 1.104 **
LDL (mg/dL)	1.438 ± 0.09801	1.815 ± 0.06538 *
VLDL (mg/dL)	9.503 ± 0.7429	13.26 ± 0.4023 **

Data are presented as mean ± SEM. Statistical differences determined by unpaired Student’s *t*-tests with GraphPad Prism 7.0. * *p*-value < 0.05, ** *p*-value < 0.01.

**Table 4:** Serum lipids analysis of Cyp2b-null and hCYP2B6-Tg mice.

Serum Parameter	Cyp2-Null Male	hCYP2B6-Tg Male
Cholesterol (mg/dL)	104.1 ± 2.863	117.1 ± 2.289 *
Triglycerides (mg/dL)	82.18 ± 4.017	132 ± 6.781 ***
HDL (mg/dL)	54.54 ± 1.709	62.77 ± 1.331 *
LDL (mg/dL)	2.938 ± 0.1074	3.305 ± 0.6967
VLDL (mg/dL)	16.44 ± 0.8024	26.4 ± 1.356 ***
**Serum Parameter**	**Cyp2-Null Female**	**hCYP2B6-Tg Female**
Cholesterol (mg/dL)	74.5 ± 1.829	77.64 ± 4.385
Triglycerides (mg/dL)	95.96 ± 4.5	67.18 ± 5.404 **
HDL (mg/dL)	37.76 ± 0.8123	38.55 ± 1.162
LDL (mg/dL)	4.736 ± 0.2744	4.72 ± 0.8014
VLDL (mg/dL)	19.19 ± 0.8999	13.44 ± 1.08 **

Data are presented as mean ± SEM. Statistical differences determined by unpaired *t*-tests using GraphPad Prism 7.0. * *p*-value < 0.05, ** *p*-value < 0.01, *** *p*-value < 0.001.

**Table 5:** DEG counts comparing transcriptome of Cyp2b-null vs. hCYP2B6-Tg mice.

Comparison	Total DEG	Enriched in hCYP2B6-Tg Mice	Enriched in Cyp2b-Null Mice
Male Cyp2b-null vs. Male hCYP2B6-Tg	807	414	393
Female Cyp2b-null vs. Female hCYP2B6-Tg	1224	623	601
Males and Females SHARED	152	58	38

*n* = 4. Differential genes determined by read counts from gene expression level analysis, followed by model dependent *p*-value estimation and FDR (adjusted *p*-value) value estimation based on multiple hypothesis testing.

**Table 6:** RNA sequencing and qPCR data for DEG in Cyp2b-null vs. hCYP2B6-Tg mice.

	Males—Fold Change		Females—Fold Change	
Gene	RNAseq	qPCR	RNAseq	qPCR
*Ppp1r3c*	−1.044 **	−0.639 *	−0.696	0.101
*Lipc*	0.556 ***	0.143	−0.725 ***	−0.704 *
*Lpin1*	1.117 **	1.294	1.528 ***	1.550 **
*Igfbp2*	0.760 *	0.616 *	−0.496	−0.668
*Plscr4*	−0.669 *	−0.893 **	0.505	0.268
*Scd1*	−1.390 ***	−1.471 ***	−1.253 *	−0.934 ***
*Plin4*	−0.775 *	−1.618 ***	−0.736 *	0.110

Data expressed as log2 fold change compared to Cyp2b-null. Significance determined by read counts from gene expression level analysis, followed by model dependent *p*-value estimation and FDR (adjusted *p*-value) value estimation based on multiple hypothesis testing. Significance determined by unpaired *t*-tests with GraphPad Prism 7.0 for qPCR. * *p*-value < 0.05, ** *p*-value < 0.01, *** *p*-value < 0.001.

## Data Availability

All data is available upon request. Supplementary Files containing quantitative data presented in this manuscript can be found in GEO (GSE282292) and *Mendeley Data*: Eccles-Miller, Jazmine; Johnson, Tyler; Baldwin, William (2024), “Sexually dimorphic effects of CYP2B6 in the development of fasting-mediated steatosis in mice: Role of the oxylipin products 9-HODE and 9-HOTrE”, Mendeley Data, V1, doi: 10.17632/wnvkz8f7ft.1—https://data.mendeley.com/datasets/wnvkz8f7ft/1 (accessed on 31 December 2024). In addition, pictures of all slides are available upon request.

## Supplementary Materials

The following supporting information can be downloaded at https://www.mdpi.com/article/10.3390/biomedicines13020295/s1, Supplementary File S1: Raw data for each biological replicate, including liver triglycerides, serum lipids, and gross anatomy (organ and total body weight). Supplementary File S2: Gross anatomy analysis to determine differences between gross anatomy of oxylipin-treated or untreated mice or Cyp2b-null and hCYP2B6-Tg mice. Supplementary File S3: RNA sequencing data analysis for 9-HODE treated Cyp2b-null mice. Supplementary File S4: RNA sequencing analysis for 9-HOTrE treated Cyp2b-null mice. Supplementary File S5: RNA sequencing data analysis for genes enriched in hCYP2B6-Tg male mice compared to Cyp2b-null male mice. Supplementary File S6: RNA sequencing data analysis for genes enriched in Cyp2b-null male mice compared to hCYP2B6-Tg male mice. Supplementary File S7: RNA sequencing data analysis for genes enriched in hCYP2B6-Tg female mice compared to Cyp2b-null female mice. Supplementary File S8: RNA sequencing data analysis for genes enriched in Cyp2b-null female mice compared to hCYP2B6-Tg female mice. Supplementary File S9: Shared DEG between male and female analysis for Cyp2b-null and hCYP2B6-Tg mice.
